# Elevated monocyte-lymphocyte ratio as an independent predictor of all-cause and cardiovascular mortality in hypertensive patients: A retrospective cohort study

**DOI:** 10.1097/MD.0000000000048243

**Published:** 2026-04-10

**Authors:** Chao Wang, Yun Gao, Bo Li, Qikeng Zhang, Zhenyan Xie, Huixi Xie, Qian Zhu, Xuesong Li

**Affiliations:** a Department of Neurosurgery, Huizhou Third People’s Hospital, The Affiliated Huizhou Hospital, Guangzhou Medical University, Huizhou, China.

**Keywords:** all-cause mortality, cardiovascular mortality, hypertensive, monocyte-to-lymphocyte ratio

## Abstract

The monocyte-to-lymphocyte ratio (MLR) has been proposed as a promising inflammatory biomarker, with potential implications for cardiovascular prognosis. However, its association with mortality in hypertensive patients remains unclear. This study aimed to investigate the correlation between MLR and all-cause and cardiovascular mortality in this population. We included 15,871 hypertensive patients from the National Health and Nutrition Examination Survey (2003–2010), with external validation using National Health and Nutrition Examination Survey (2011–2018) data. Mortality details were obtained from the National Death Index. Weighted multivariable Cox regression evaluated the independent association between MLR and mortality risk; restricted cubic spline analysis visualized the relationship, and receiver operating characteristic curves assessed predictive performance. Over a median follow-up of 141 months, 2999 all-cause deaths (18.9%) and 956 cardiovascular deaths (6.0%) occurred. Using an MLR threshold of 0.39, participants were stratified into higher (>0.39) and lower (≤0.39) groups. Fully adjusted models showed that elevated MLR significantly increased all-cause mortality risk (hazard ratio = 1.42, 95% confidence interval: 1.29–1.50, *P* < .0001) and cardiovascular mortality risk (hazard ratio = 1.59, 95% confidence interval: 1.36–1.80, *P* < .0001). Restricted cubic spline revealed a U-shaped association (*P* < .05). External validation confirmed that higher MLR correlated with increased mortality. The area under the curve for 1-, 3-, 5-, and 10-year survival was 0.73, 0.69, 0.67, and 0.66 for all-cause mortality and 0.70, 0.69, 0.71, and 0.68 for cardiovascular mortality, respectively. Elevated MLR is independently associated with higher all-cause and cardiovascular mortality risks in hypertensive patients.

## 1. Introduction

Hypertension is a worldwide health concern, with a prevalence rate of up to 40% and a low control rate, causing a significant impact on the global disease burden.^[1,2]^ Despite the extensive knowledge about the management of hypertension, its global prevalence remains high and is on the rise.^[3]^ Being a significant risk factor for cardiovascular diseases and all-cause mortality,^[4]^ hypertension poses a substantial challenge to clinicians and public health. Therefore, identifying the potential factors that contribute to the increased all-cause and cardiovascular mortality in hypertensive patients is crucial for developing effective preventive measures and reducing mortality rates.

Research has shown that inflammation is strongly linked to the onset and development of hypertension, cardiovascular and cerebrovascular diseases, and significantly impacts the mortality of chronic diseases.^[5–7]^ Against this backdrop, the relationship between various inflammatory markers and related diseases has become a research focus. For instance, studies have indicated that the neutrophil-to-lymphocyte ratio (NLR), as an indicator of systemic inflammation, can predict the risk of mortality in hypertensive patients.^[8]^ The monocyte-to-lymphocyte ratio (MLR) refers to the ratio of the number of monocytes to the number of lymphocytes in peripheral blood. MLR can be determined using routine blood test parameters and effectively reflects the body’s overall inflammation level and immune response. Monocytes penetrate the endothelium and differentiate into resident macrophages, accumulating in the arterial wall and activating the secretion of pro-inflammatory cytokines, matrix metalloproteinases, and reactive oxygen species. These processes not only contribute to low-grade inflammatory responses but also play a critical role in the formation and rupture of atherosclerotic plaques, thereby driving the onset and progression of hypertension and cardiovascular and cerebrovascular diseases.^[9–11]^ Lymphocytes play a critical role in the pathology of hypertension and cardiovascular injury by regulating inflammation and immune responses. Pro-inflammatory lymphocytes, such as Th1, Th17, and γδ T cells, exacerbate inflammatory responses by secreting pro-inflammatory cytokines, leading to vascular dysfunction and damage, thereby promoting the development and progression of hypertension and cardiovascular diseases.^[12]^ By contrast, anti-inflammatory regulatory T cells exert a protective effect by suppressing pro-inflammatory responses.^[12]^ Given the pivotal role of these immune cells in disease progression, researchers have increasingly focused on the MLR as a novel inflammatory marker to better understand its role in disease and prognosis. Previous research indicates that higher MLR levels are associated with an increased risk of all-cause and cardiovascular mortality in patients with chronic kidney disease^[13]^ and diabetes.^[14]^ However, to our knowledge, no research has thoroughly examined the link between MLR and mortality risk in hypertensive patients. Therefore, this study seeks to explore the relationship between MLR and the risk of all-cause and cardiovascular mortality in hypertensive patients.

Thus, we hypothesize that higher MLR levels could be linked to an increased risk of mortality (including all-cause and cardiovascular) in hypertensive patients. To test this hypothesis, we will analyze data from the National Health and Nutrition Examination Survey (NHANES) 2003 to 2010 and use NHANES 2011 to 2018 data for external validation. If we can confirm an association between MLR and mortality rates in hypertensive patients, this could contribute to improving risk stratification and management strategies for these patients, potentially leading to a reduction in hypertension-related mortality.

## 2. Methods

### 2.1. Data source and study population

The NHANES database consists of 5 components: questionnaires, laboratory tests, exams, food intake, and demographics. It provides comprehensive information on the health and nutritional condition of the American demographic. Prior to data collection, the National Center for Health Statistics’ ethical oversight commission secured informed consent from all participants. The program is regularly updated, with the newest survey data made available every 2 years for researchers. Data from the NHANES database for the years 2003 to 2010 were used in the study. Of the 41,556 participants, 15,871 were included in this retrospective cross-sectional analysis after excluding participants aged <20 years (n = 18,983), those without hypertension (n = 1313), those lacking monocyte or lymphocyte data (n = 1391), those without follow-up data (n = 24), and those with missing covariate data (n = 3574). A complete-case analysis was therefore performed.

For external validation, we extracted data from NHANES 2011 to 2018, applying the same exclusion criteria as for the 2003 to 2010 cohort, ultimately including 15,344 participants in the validation analysis.

### 2.2. Definition of hypertension and measurement of MLR

The definition of hypertension includes any of the following: a self-reported history of hypertension, the use of blood pressure medication, a blood pressure average of at least 140 mm Hg at the systolic, and/or 90 mm Hg at the diastolic levels. NHANES staff measured blood samples from participants at mobile examination centers using the Beckman Coulter counting and sizing method. This process provided complete blood count parameters, including monocyte and lymphocyte counts. By dividing the number of monocytes by the number of lymphocytes, the MLR was computed.

### 2.3. Ascertainment of mortality and follow-up

The research examined all-cause mortality and cardiovascular mortality in hypertensive patients. Mortality data were sourced from the National Death Index database maintained by the US Centers for Disease Control and Prevention (https://www.cdc.gov/nchs/linked-data/mortality-files/index.html). For each participant, the follow-up period extended from the date of enrollment until either the mortality date or December 31, 2019 (the last updated time of the National Death Index database). Cardiovascular disease mortality was defined as deaths caused by cerebrovascular diseases or cardiovascular diseases, identified using the International Classification of Diseases, 10th Revision codes I00-I09, I11, I13, I20-I51, and I60-I69.^[13]^

### 2.4. Covariates and defining disease

Continuous variables included age, body mass index, white blood cell count, total cholesterol, high-density lipoprotein, neutrophils, blood urea nitrogen (BUN), serum creatinine (Scr), aspartate aminotransferase, and alanine aminotransferase. Categorical variables include sex, race (Mexican American, non-Hispanic White, non-Hispanic Black, and other race), marital status (unmarried, divorced/separated, widowed, or married), education levels (<high school, high school graduate, or >high school), smoking status, drinking status, moderate-intensity physical activity (MIPA), anemia (non-anemia, mild, moderate, and severe), diabetes, and poverty-income ratio (PIR). These variables were included as covariates. The diagnostic criteria for diabetes are met if an individual has any 1 or more of the following: a physician’s diagnosis of diabetes; glycosylated hemoglobin ≥6.5%; or the use of insulin or diabetes medications.^[15]^ The PIR was divided into 3 different groups: <1.5, between 1.5 and 3.5, and >3.5.^[16]^ Smoking status was categorized as never smokers (fewer than 100 cigarettes in a lifetime), former smokers (more than 100 cigarettes but quit), and current smokers (more than 100 cigarettes and still smoking).^[17]^ The following categories are used to classify drinking^[17]^: never drinkers: have drunk <12 times in their lives. Former drinkers: have drunk more than 12 times in their lives but have not drunk in the past year. Heavy drinkers are women who drink ≥3 glasses of alcohol per day or men who drink ≥4 glasses of alcohol per day, and who drink on ≥5 days per month. Moderate drinkers are women who drink ≥2 glasses of alcohol per day or men who drink ≥3 glasses of alcohol per day, and who drink on 2 to 4 days per month. Light drinkers do not satisfy the criteria for being classified as moderate or heavy drinkers. Lastly, according to the NHANES Physical Activity Questionnaire, MIPA was defined as any activity that induces slight sweating or a moderate increase in heart rate and breathing rate, lasting a minimum of 10 minutes.

### 2.5. Statistical analysis

In this study, we utilized the full sample 2-year MEC exam weight from NHANES to represent the entire national population. Before data analysis, we tested the continuous variables for normality and found that they were all non-normally distributed. Therefore, we represented them using the median (interquartile range). Categorical variables were shown as frequencies and percentages. For continuous variables, we used either Student’s *t* test or the nonparametric Mann–Whitney *U* test. For categorical variables, we applied either the chi-square test or the Fisher exact test. The optimal cutoff value of MLR was determined to be 0.39 using the “Survminer” R package based on the overall study population. Using this cutoff, participants were categorized into a higher MLR group (>0.39) and a lower MLR group (≤0.39). The relationship between MLR and all-cause mortality and cardiovascular mortality in hypertensive patients was assessed using weighted Cox regression analysis. The crude model was unadjusted for covariates. Model 1 was adjusted for age, sex, and race. Model 2 included additional adjustments for marital status, education, PIR, body mass index, total cholesterol, smoking, drinking, diabetes, Scr, BUN, aspartate aminotransferase, and neutrophils. The potential nonlinear association between MLR and cardiovascular and all-cause mortality in hypertensive individuals was depicted using the restricted cubic spline with 4 knots. Kaplan–Meier survival analysis was employed to compare the survival rates between the higher MLR group and the lower MLR group, with the log-rank test employed for statistical comparison. A time-dependent receiver operating characteristic (ROC) curve analysis was carried out to evaluate the performance of the MLR in forecasting survival results at various time points, and the area under the curve (AUC) was calculated. To validate the association between the MLR and both all-cause and cardiovascular mortality in hypertensive patients, we conducted 3 sensitivity analyses. First, we performed a subgroup analysis, examining the relationship between MLR levels and mortality across various demographic and health factors, including age, gender, smoking and drinking profile, diabetes, anemia, and MIPA. Second, to mitigate potential reverse causality, we excluded participants who died within 3 years of the initial interview from our analysis. Finally, we employed an alternative threshold analysis, using 0.8 as an optimal MLR threshold to assess the robustness of our findings across different cut-points.

Similarly, in the external validation analysis, participants were also divided into a higher MLR group (>0.39) and a lower MLR group (≤0.39). The following analyses were conducted: weighted Cox regression analyses were performed to validate the association between MLR levels and both all-cause and cardiovascular mortality in hypertensive patients, using the same covariate adjustments as in the primary Cox regression models; time-dependent ROC curve analyses were conducted at 1, 3, and 5 years to assess the predictive performance of MLR, with the AUC calculated at each time point, as the follow-up duration in the external dataset was <10 years; sensitivity analyses were also performed to further examine the robustness of the results. The data analysis and figure design were performed using R version 4.3.2 (R Foundation for Statistical Computing, Vienna, Austria) in RStudio (Posit Software, PBC, Boston). *P* < .05 was considered significant.

## 3. Results

### 3.1. Participant characteristics

**Figure 1.**
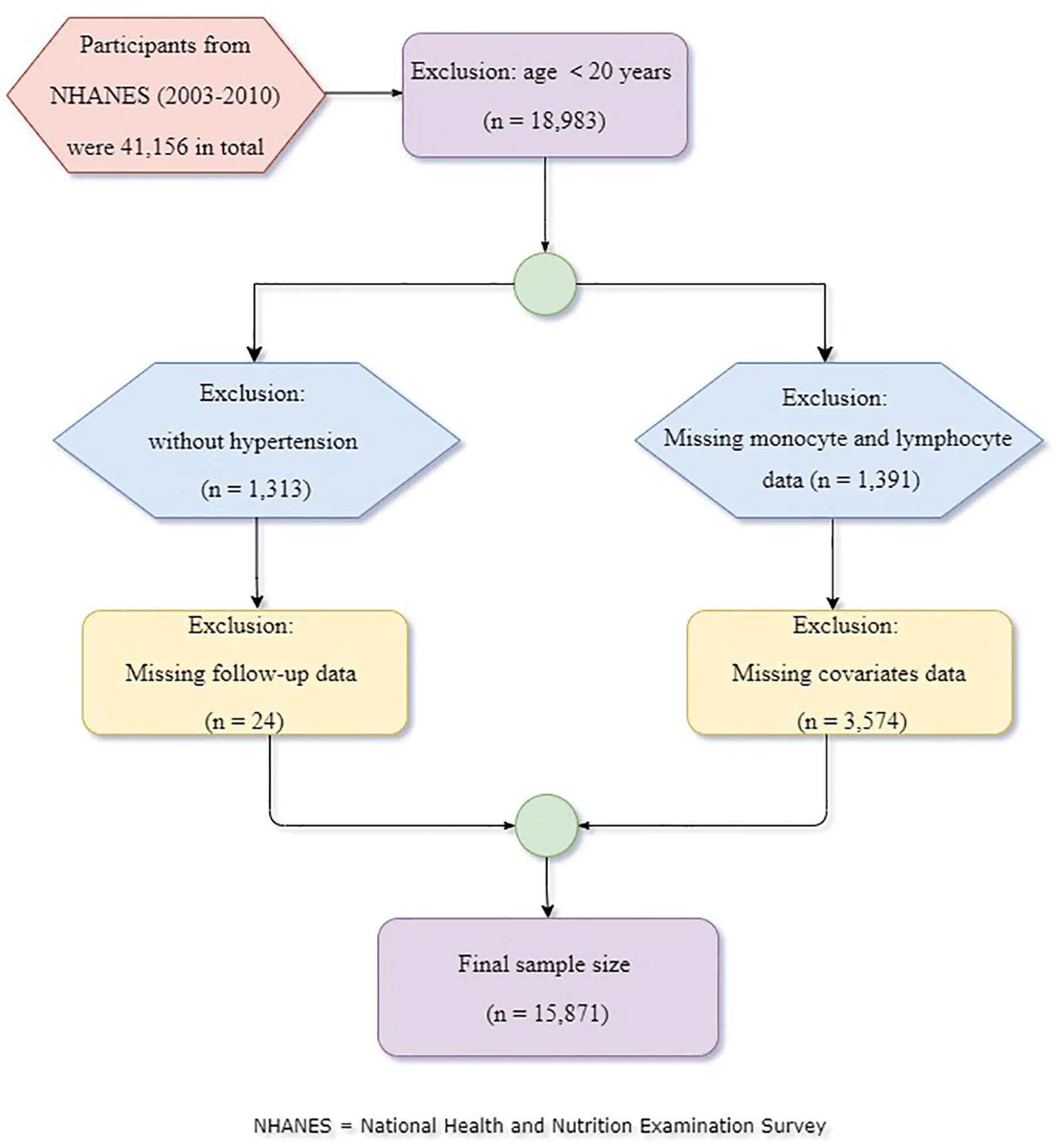
The flow diagram for participant selection. NHANES = National Health and Nutrition Examination Survey.

**Figure 2.**
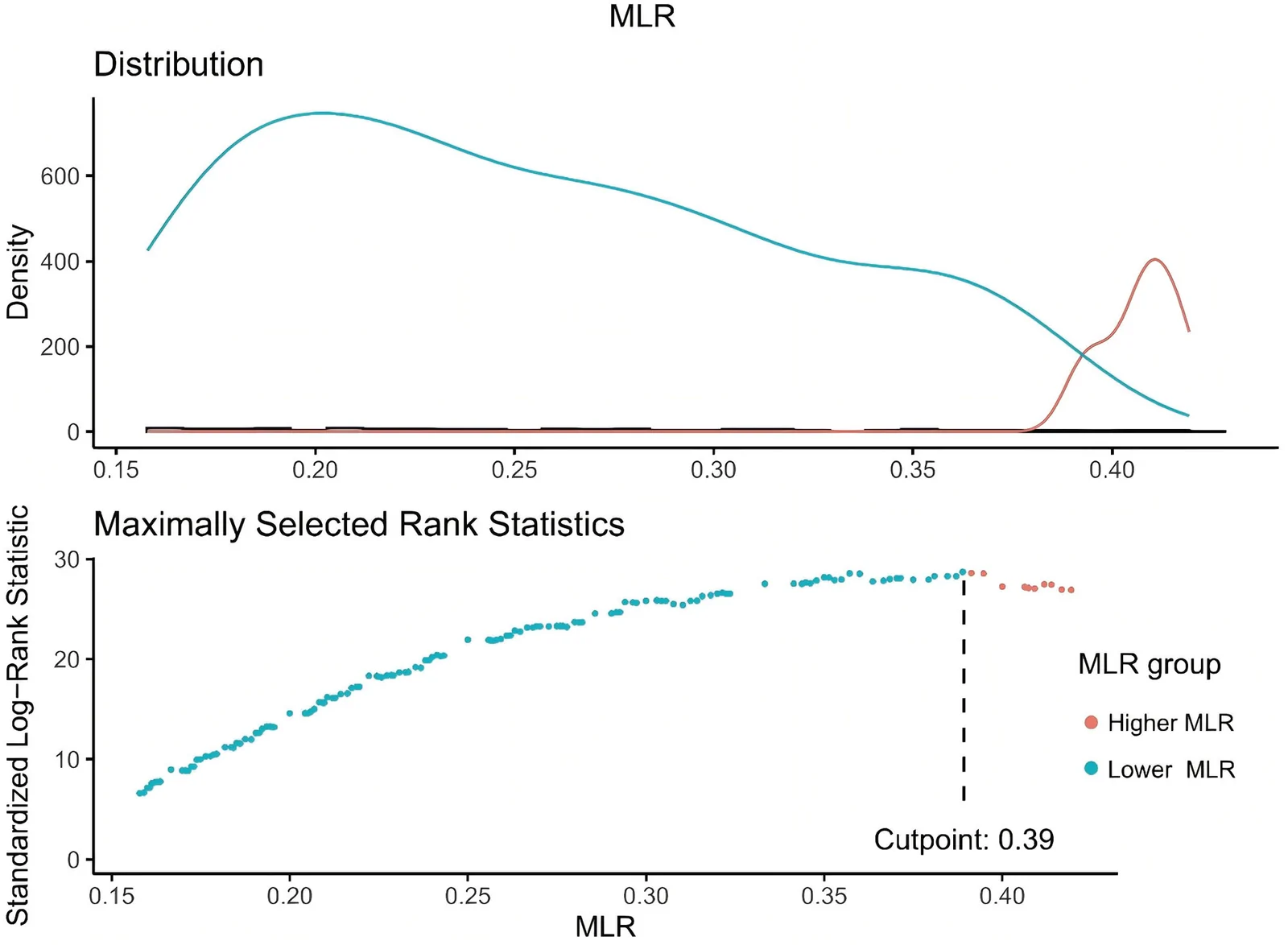
The optimal cutoff point for MLR. MLR = monocyte-to-lymphocyte ratio.

The flow diagram for participant selection is shown in Figure 1. This study included 15,871 NHANES participants, representing 166,355,774 hypertensive individuals in the United States. Utilizing the optimal MLR cutoff value of 0.39, identified as having the strongest correlation with survival based on maximally selected rank statistics method, participants were categorized into higher (MLR > 0.39) and lower (MLR ≤ 0.39) groups (Fig. 2). The weighted baseline characteristics of these participants are shown in Table 1. Specifically, participants in the higher MLR group were older than those in the lower MLR group and had a greater proportion of non-Hispanic White males. They also showed elevated levels of Scr, BUN, high-density lipoprotein, and neutrophil counts, as well as higher rates of anemia and diabetes.

**Table 1:** Characteristics of study population.

Variable	Total (n = 15871)	Higher MLR (n = 2157)	Lower MLR (n = 13714)	*P* value
Age, yr	50.0 (35.0–65.0)	65.0 (46.0–77.0)	48.0 (34.0–63.0)	<.0001
Sex, %				<.0001
Female	7780 (49.0%)	720 (33.4%)	7060 (51.5%)	
Male	8091 (51.0%)	1437 (66.6%)	6654 (48.5%)	
Race, %				<.0001
Mexican American	2912 (18.3%)	222 (10.3%)	2690 (19.6%)	
Non-Hispanic Black	3013 (19.0%)	276 (12.8%)	2737 (20.0%)	
Non-Hispanic White	8257 (52.0%)	1496 (69.4%)	6761 (49.3%)	
Other race	1689 (10.6%)	163 (7.56%)	1526 (11.1%)	
BMI, kg/m^2^	27.9 (24.4–32.2)	27.1 (23.8–31.1)	28.1 (24.4–32.4)	<.0001
Education levels, %				.0022
<High school	4408 (27.8%)	562 (26.1%)	3846 (28.0%)	
High school	3827 (24.1%)	583 (27.0%)	3244 (23.7%)	
>High school	7636 (48.1%)	1012 (46.9%)	6624 (48.3%)	
Marital status, %				<.0001
Divorced/separated	2212 (13.9%)	284 (13.2%)	1928 (14.1%)	
Married	9738 (61.4%)	1288 (59.7%)	8450 (61.6%)	
Unmarried	2487 (15.7%)	252 (11.7%)	2235 (16.3%)	
Widowed	1434 (9.04%)	333 (15.4%)	1101 (8.03%)	
PIR, %				<.0001
<1.5	5480 (34.5%)	656 (30.4%)	4824 (35.2%)	
1.5–3.5	5289 (33.3%)	799 (37.0%)	4490 (32.7%)	
≥3.5	5102 (32.1%)	702 (32.5%)	4400 (32.1%)	
Smoking status, %				<.0001
Never	8110 (51.1%)	978 (45.3%)	7132 (52.0%)	
Former	4195 (26.4%)	801 (37.1%)	3394 (24.7%)	
Now	3566 (22.5%)	378 (17.5%)	3188 (23.2%)	
Drinking status, %				<.0001
Never	2095 (13.2%)	259 (12.0%)	1836 (13.4%)	
Former	3255 (20.5%)	516 (23.9%)	2739 (20.0%)	
Mild	5001 (31.5%)	770 (35.7%)	4231 (30.9%)	
Moderate	2278 (14.4%)	243 (11.3%)	2035 (14.8%)	
Heavy	3242 (20.4%)	369 (17.1%)	2873 (20.9%)	
Diabetes, %				.0007
No	13,534 (85.3%)	1787 (82.8%)	11,747 (85.7%)	
Yes	2337 (14.7%)	370 (17.2%)	1967 (14.3%)	
Anemia, %				<.0001
Non-anemic	14,673 (92.5%)	1882 (87.3%)	12,791 (93.3%)	
Mild	896 (5.65%)	207 (9.60%)	689 (5.02%)	
Moderate	294 (1.85%)	65 (3.01%)	229 (1.67%)	
Severe	8 (0.05%)	3 (0.14%)	5 (0.04%)	
MIPA, %				.0004
No	7775 (49.0%)	1134 (52.6%)	6641 (48.4%)	
Yes	8096 (51.0%)	1023 (47.4%)	7073 (51.6%)	
Scr, μmol/L	79.6 (64.5–89.3)	85.8 (70.7–99.0)	76.9 (63.6–88.4)	<.0001
BUN, mmol/L	4.28 (3.57–5.71)	5.00 (3.93–6.78)	4.28 (3.57–5.36)	<.0001
Neutrophils, ×10^9^/L	4.00 (3.10–5.10)	4.40 (3.50–5.60)	4.00 (3.10–5.00)	<.0001
WBC, ×10^9^/L	6.90 (5.70–8.30)	6.90 (5.60–8.40)	6.90 (5.70–8.30)	.2226
TC, mg/dL	194 (168–223)	186 (160–214)	196 (170–224)	<.0001
HDL, mg/dL	50.0 (41.0–62.0)	51.0 (42.0–63.0)	50.0 (41.0–62.0)	.0014
ALT, U/L	21.0 (17.0–29.0)	21.0 (17.0–28.0)	21.0 (17.0–29.0)	.0400
AST, U/L	23.0 (20.0–28.0)	24.0 (21.0–29.0)	23.0 (20.0–28.0)	<.0001

ALT = alanine aminotransferase, AST = aspartate aminotransferase, BMI = body mass index, BUN = blood urea nitrogen, HDL = high-density lipoprotein, MIPA = moderate-intensity physical activity, MLR = monocyte-to-lymphocyte ratio, PIR = poverty-income ratio, Scr = serum creatinine, TC = total cholesterol, WBC = white blood cell.

### 3.2. The relationship between mortality and MLR

**Figure 3.**
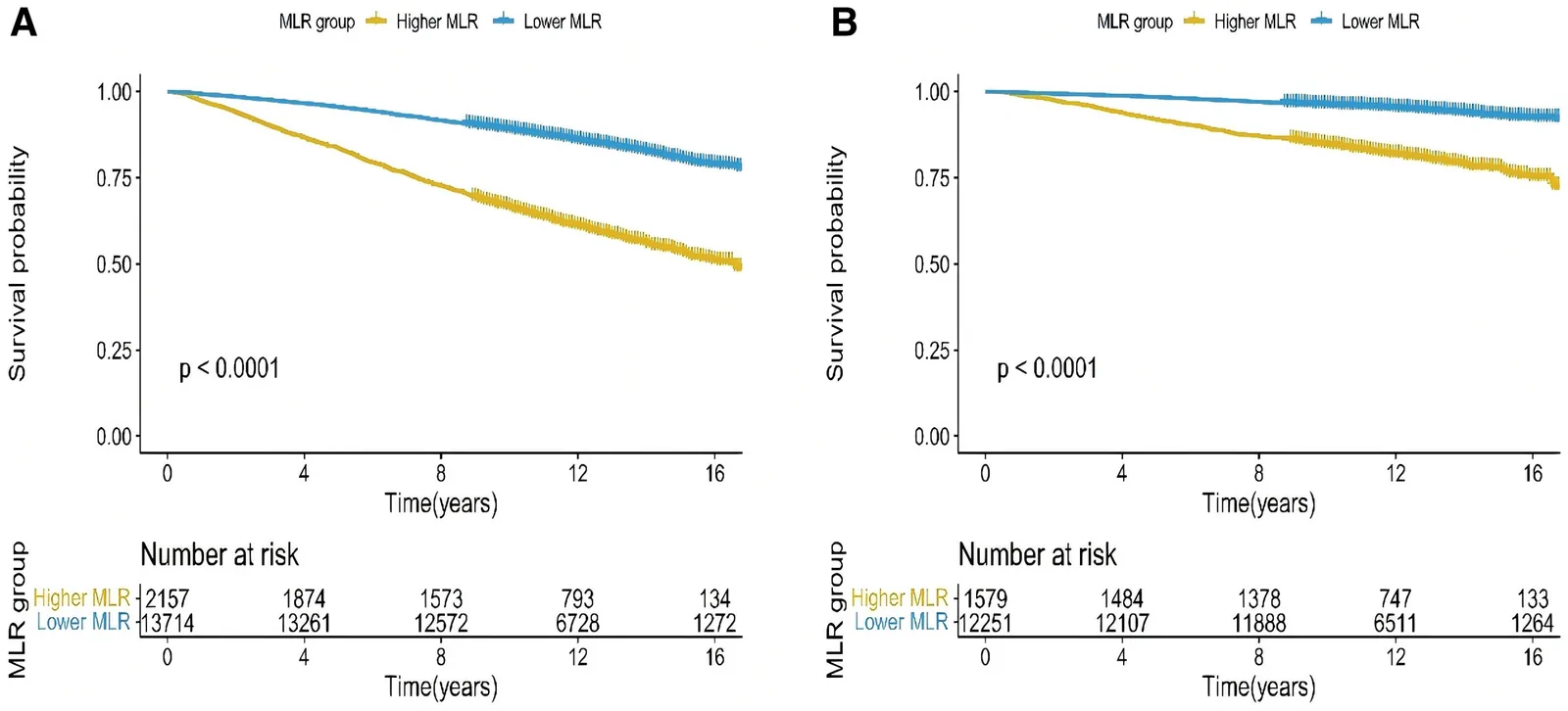
Kaplan–Meier curves illustrating the survival rates of hypertensive patients with higher (>0.39) versus lower (≤0.39) MLR values. (A) All-cause mortality; (B) cardiovascular mortality. MLR = monocyte-to-lymphocyte ratio.

The results of the weighted Cox regression analysis in Table 2 reveal a substantial link between MLR and the risk of all-cause mortality. In the unadjusted crude model, this association is particularly strong (hazard ratio [HR] = 16.69, 95% confidence interval [CI]: 11.24–24.7, *P* < .0001). Even after multivariable adjustment in model 1 (HR = 3.57, 95% CI: 2.83–4.50, *P* < .0001) and model 2 (HR = 3.24, 95% CI: 2.53–4.10, *P* < .0001), this correlation remains noteworthy. The higher MLR group exhibits a markedly increased risk of all-cause mortality compared to the lower MLR group. This result is evident across all models, from the crude (HR = 3.18, 95% CI: 2.90–3.47, *P* < .0001) to the adjusted ones (model 1: HR = 1.53, 95% CI: 1.39–1.60, *P* < .0001; model 2: HR = 1.42, 95% CI: 1.29–1.50, *P* < .0001). Similarly, the association between MLR and the risk of cardiovascular disease mortality is also very significant. In the crude model, MLR has a higher HR (HR = 23.91, 95% CI: 14.39–39.70, *P* < .0001), and this correlation remains notable after multivariable adjustment in model 1 (HR = 5.54, 95% CI: 3.97–7.70, *P* < .0001) and model 2 (HR = 5.49, 95% CI: 3.84–7.80, *P* < .0001). Like the results for all-cause mortality, the high MLR group has a significantly increased risk of cardiovascular mortality compared to the low MLR group, from the crude model (HR = 4.17, 95% CI: 3.56–4.87, *P* < .0001) to the adjusted model 2 (HR = 1.59, 95% CI: 1.36–1.80, *P* < .0001), all showing significant statistical significance. Figure 3 displays the Kaplan–Meier survival curves for all-cause and cardiovascular mortality in hypertensive participants, stratified by MLR groups. The findings show that participants in the higher MLR group exhibit significantly increased rates of both all-cause and cardiovascular mortality relative to those in the lower MLR group (*P* < .0001).

**Table 2:** Relationship between MLR and mortality in hypertensive patients.

Characteristic	Crude model		Model 1		Model 2	
	HR (95% CI)	*P*	HR (95% CI)	*P*	HR (95% CI)	*P*
All-cause mortality						
MLR	16.69 (11.24–24.7)	<.0001	3.57 (2.83–4.51)	<.0001	3.24 (2.53–4.14)	<.0001
MLR category						
Lower MLR	Ref	Ref	Ref	Ref	Ref	Ref
Higher MLR	3.18 (2.90–3.47)	<.0001	1.53 (1.39–1.69)	<.0001	1.42 (1.29–1.56)	<.0001
Cardiovascular mortality						
MLR	23.91 (14.39–39.7)	<.0001	5.54 (3.97–7.72)	<.0001	5.49 (3.84–7.86)	<.0001
MLR category						
Lower MLR	Ref	Ref	Ref	Ref	Ref	Ref
Higher MLR	4.17 (3.56–4.87)	<.0001	1.73 (1.48–2.04)	<.0001	1.59 (1.36–1.85)	<.0001

Crude model was unadjusted for covariates.

Model 1 was adjusted for age, sex, and race.

Model 2 was further adjusted for age, sex, race, marital status, education levels, poverty-income ratio (PIR), body mass index (BMI), total cholesterol (TC), smoking status, drinking status, diabetes, serum creatinine (Scr), blood urea nitrogen (BUN), aspartate aminotransferase (AST), and neutrophils.

CI = confidence interval, HR = hazard ratio, MLR = monocyte-to-lymphocyte ratio.

### 3.3. Time-dependent ROC analysis

**Figure 4.**
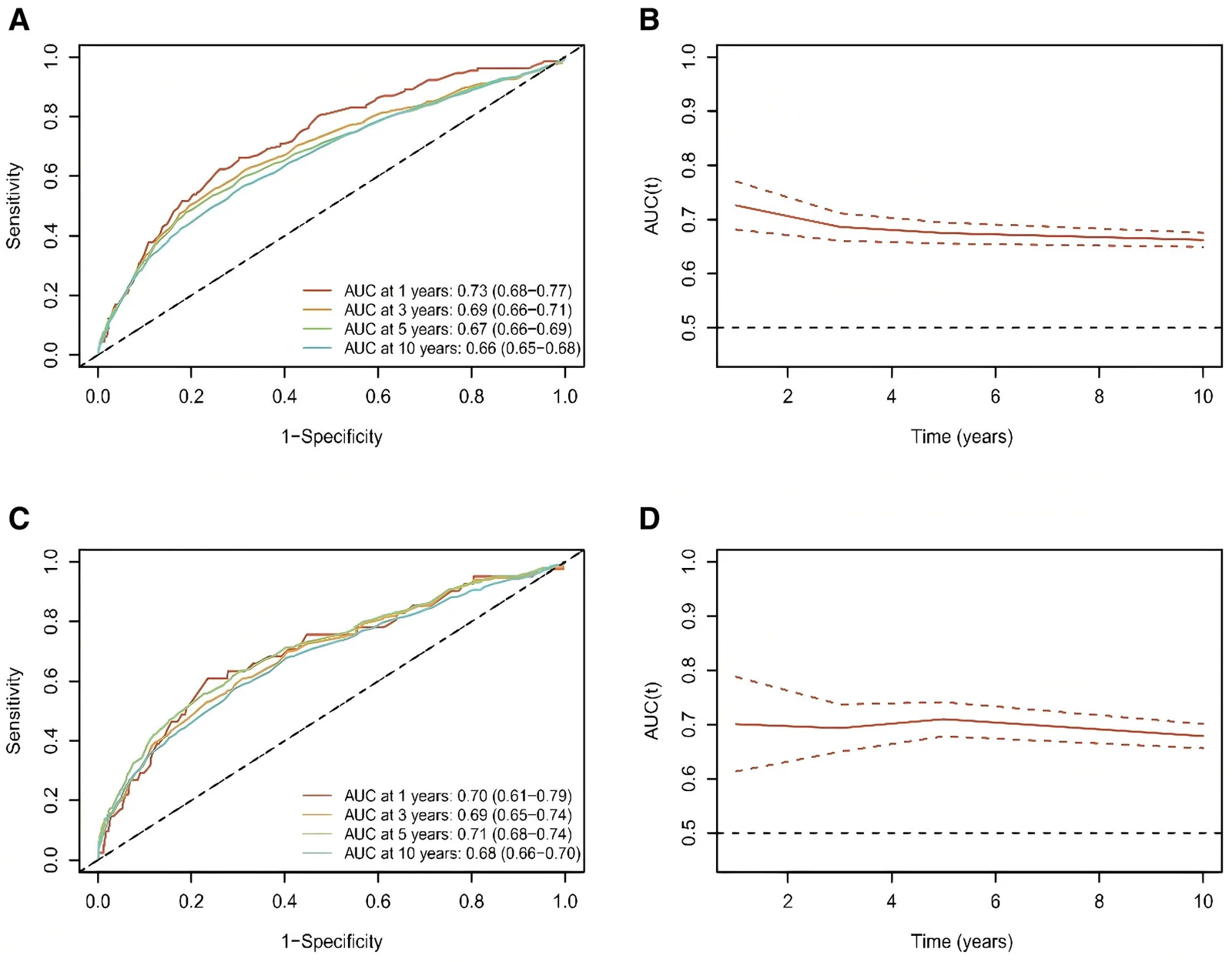
Time-dependent ROC curves of the MLR for predicting all-cause mortality (A, B) and cardiovascular mortality (C, D). AUC = area under the curve, MLR = monocyte-to-lymphocyte ratio, ROC = receiver operating characteristic.

We conducted a time-dependent ROC analysis to evaluate the capability of MLR in predicting mortality (all-cause and cardiovascular) in hypertensive patients. This approach assessed the prognostic value of MLR at different time points throughout the follow-up period. The results revealed that the AUCs for MLR in forecasting all-cause mortality at 1, 3, 5, and 10 years were 0.73 (95% CI: 0.68–0.77), 0.69 (95% CI: 0.66–0.71), 0.67 (95% CI: 0.66–0.69), and 0.66 (95% CI: 0.65–0.68) in that order (Fig. 4A and B). For predicting cardiovascular mortality at 1, 3, 5, and 10 years, the AUCs of MLR were 0.70 (95% CI: 0.61–0.79), 0.69 (95% CI: 0.65–0.74), 0.71 (95% CI: 0.68–0.74), and 0.68 (95% CI: 0.66–0.70), respectively (Fig. 4C and D). In summary, the time-dependent ROC analysis indicates that MLR is a significant predictor of both all-cause and cardiovascular mortality in hypertensive patients. The AUC values demonstrate the effectiveness of MLR as a prognostic marker at different time intervals.

### 3.4. Dose-response relationship between MLR and mortality

**Figure 5.**
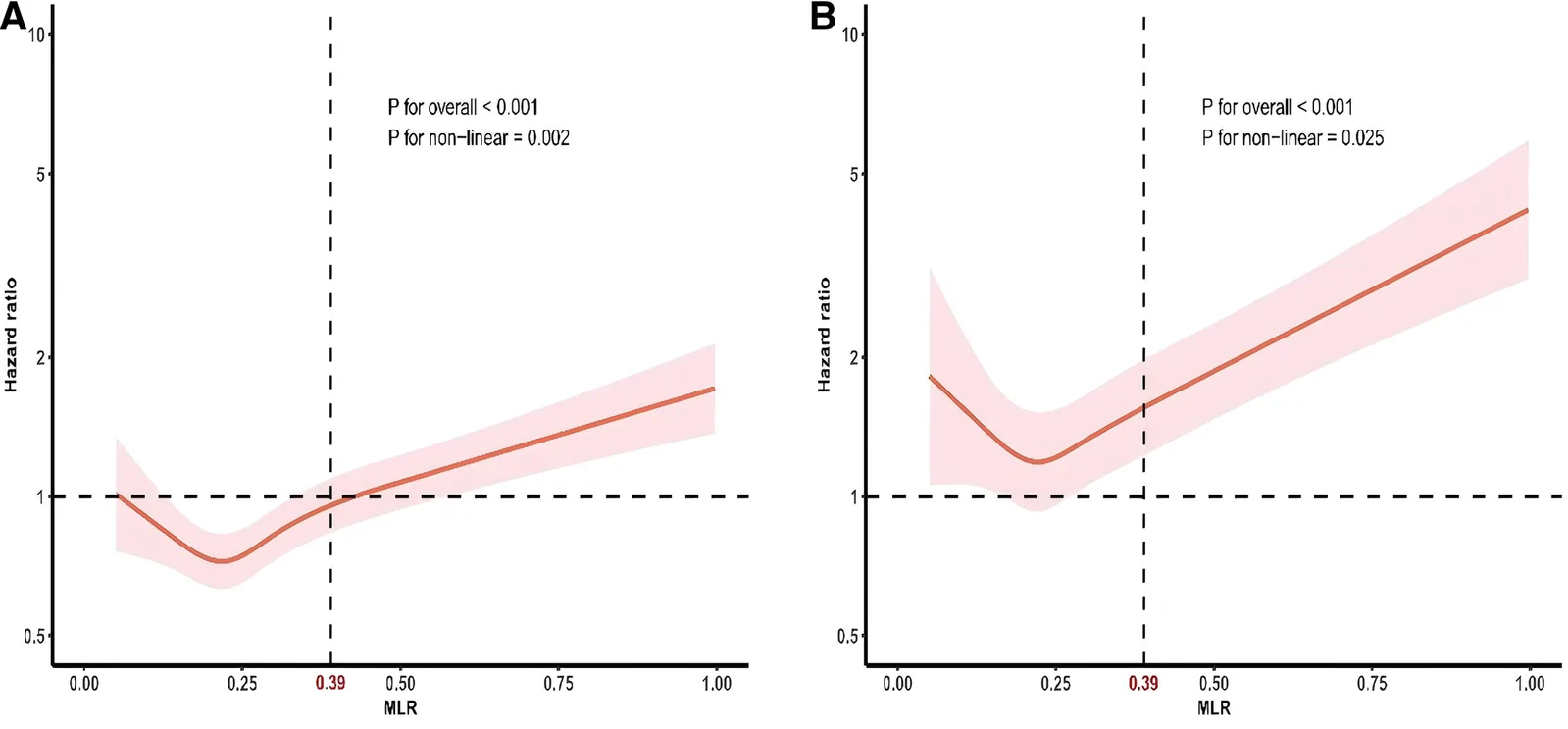
Restricted cubic spline curves of relations between MLR with all-cause (A) and cardiovascular mortality (B). Analysis was adjusted for age, sex, race, marital status, education levels, PIR, BMI, TC, smoking status, drinking status, diabetes, Scr, BUN, AST, and neutrophils. AST = aspartate aminotransferase, BMI = body mass index, BUN = blood urea nitrogen, MLR = monocyte-to-lymphocyte ratio, PIR = poverty-income ratio, Scr = serum creatinine, TC = total cholesterol.

We performed restricted cubic spline analysis to clarify the link between MLR and mortality rates (Fig. 5). After adjusting for potential confounding factors, we observed distinct U-shaped relationships between MLR and both all-cause mortality (nonlinear *P* = .002, overall *P* < .001) and cardiovascular disease mortality (nonlinear *P* = .028, overall *P* < .001). The mortality risk reached its nadir at an MLR of approximately 0.25. When the MLR exceeded 0.39, there was a marked increase in mortality risk. It is particularly noteworthy that as the MLR approached 1, the HR substantially surpassed the baseline, indicating a significantly elevated risk of death at higher MLR values. This U-shaped pattern indicates that both low and high MLR levels are associated with increased mortality risk, whereas an intermediate MLR level confers the lowest risk.

### 3.5. Sensitivity analysis

Subgroup analysis: the results demonstrated that higher MLR was significantly associated with increased all-cause and cardiovascular mortality across all subgroups (Tables 3 and 4). Importantly, no significant interactions were observed between MLR and key demographic variables, including age and sex (all *P* for interaction > .05), indicating that the prognostic effect of MLR was stable across these subgroups. Notably, significant interactions between MLR and anemia status were observed in the all-cause mortality analysis (*P* for interaction = .002). Exclusion of early deaths: following the exclusion of participants who passed away within 3 years of the initial interview, the link between MLR and both all-cause and cardiovascular mortality remained unchanged (Table S1, Supplemental Digital Content, https://links.lww.com/MD/R668). Alternative threshold analysis: when the optimal threshold for MLR was adjusted to 0.8, the impact of MLR on all-cause and cardiovascular mortality persisted and remained statistically significant (Table S2, Supplemental Digital Content, https://links.lww.com/MD/R668). These results strongly demonstrate the stability and reliability of the relationship between MLR and mortality in the hypertensive population.

**Table 3:** Subgroup analysis of the relationship between MLR and all-cause mortality in hypertensive patients.

Characteristic	Lower MLR (≤0.39)	Higher MLR HR (95% CI)	*P*	*P* for interaction
Age, yr				.744
<60	1	1.726 (1.384–2.151)	<.0001	
≥60	1	1.637 (1.471–1.821)	<.0001	
Sex				.133
Female	1	1.903 (1.555–2.330)	<.0001	
Male	1	2.224 (1.915–2.583)	<.0001	
Smoking status				.438
Never	1	2.119 (1.771–2.535)	<.0001	
Former/now	1	2.216 (1.953–2.515)	<.0001	
Drinking status				.076
Never	1	1.947 (1.508–2.514)	<.0001	
Former/mild/moderate/heavy	1	2.244 (1.993–2.528)	<.0001	
Diabetes				.093
No	1	2.185 (1.923–2.482)	<.0001	
Yes	1	2.044 (1.629–2.566)	<.0001	
MIPA				.848
No	1	2.151 (1.878–2.464)	<.0001	
Yes	1	2.174 (1.822–2.594)	<.0001	
Anemia				.002
No	1	2.194 (1.951–2.468)	<.0001	
Yes	1	1.852 (1.449–2.367)	<.0001	

HRs were adjusted for age, sex, race, marital status, education levels, poverty-income ratio (PIR), body mass index (BMI), total cholesterol (TC), smoking status, drinking status, diabetes, serum creatinine (Scr), blood urea nitrogen (BUN), aspartate aminotransferase (AST), and neutrophils.

CI = confidence interval, HR = hazard ratio, MIPA = moderate-intensity physical activity, MLR = monocyte-to-lymphocyte ratio.

**Table 4:** Subgroup analysis of the relationship between MLR and cardiovascular mortality in hypertensive patients.

Characteristic	Lower MLR (≤0.39)	Higher MLR HR (95% CI)	*P*	*P* for interaction
Age, yr				.185
<60	1	2.906 (1.835–4.601)	<.0001	
≥60	1	1.868 (1.576–2.215)	<.0001	
Sex				.15
Female	1	2.325 (1.621–3.335)	<.0001	
Male	1	2.844 (2.244–3.604)	<.0001	
Smoking status				.522
Never	1	2.407 (1.776–3.262)	<.0001	
Former/now	1	3.020 (2.317–3.936)	<.0001	
Drinking status				.423
Never	1	2.215 (1.386–3.540)	<.001	
Former/mild/moderate/heavy	1	2.980 (2.499–3.554)	<.0001	
Diabetes				.33
No	1	2.838 (2.315–3.479)	<.0001	
Yes	1	2.590 (1.839–3.649)	<.0001	
MIPA				.314
No	1	2.611 (2.061–3.307)	<.0001	
Yes	1	3.046 (2.293–4.046)	<.0001	
Anemia				.82
No	1	2.665 (2.156–3.295)	<.0001	
Yes	1	2.624 (1.676–4.106)	<.0001	

HRs were adjusted for age, sex, race, marital status, education levels, poverty-income ratio (PIR), body mass index (BMI), total cholesterol (TC), smoking status, drinking status, diabetes, serum creatinine (Scr), blood urea nitrogen (BUN), aspartate aminotransferase (AST), and neutrophils.

CI = confidence interval, HR = hazard ratio, MIPA = moderate-intensity physical activity, MLR = monocyte-to-lymphocyte ratio.

### 3.6. External validation of the results

For external validation, we extracted NHANES 2011 to 2018 data and constructed a weighted Cox regression model identical to that used in the primary analysis, including the same covariate adjustments. The results (Table 5) indicated that MLR was significantly associated with both all-cause mortality and cardiovascular mortality in individuals with hypertension. These findings strongly support the generalizability and robustness of our initial results.

**Table 5:** External validation analysis of the association between MLR and mortality in hypertensive patients.

Characteristic	Crude model		Model 1		Model 2	
	HR (95% CI)	*P*	HR (95% CI)	*P*	HR (95% CI)	*P*
All-cause mortality						
MLR	9.02 (4.63–17.56)	<.0001	3.84 (2.54–5.80)	<.0001	3.54 (2.51–4.98)	<.0001
MLR category						
Lower MLR	Ref	Ref	Ref	Ref	Ref	Ref
Higher MLR	3.51 (2.88–4.27)	<.0001	1.68 (1.34–2.11)	<.0001	1.53 (1.24–1.90)	<.001
Cardiovascular mortality						
MLR	11.54 (5.32–25.03)	<.0001	5.75 (3.26–10.1)	<.0001	6.43 (3.82–10.9)	<.0001
MLR category						
Lower MLR	Ref	Ref	Ref	Ref	Ref	Ref
Higher MLR	4.50 (3.32–6.12)	<.0001	1.85 (1.33–2.59)	<.001	1.70 (1.24–2.34)	.001

Crude model was unadjusted for covariates.

Model 1 was adjusted for age, sex, and race.

Model 2 was further adjusted for age, sex, race, marital status, education levels, poverty-income ratio (PIR), body mass index (BMI), total cholesterol (TC), smoking status, drinking status, diabetes, serum creatinine (Scr), blood urea nitrogen (BUN), aspartate aminotransferase (AST), and neutrophils.

CI = confidence interval, HR = hazard ratio, MLR = monocyte-to-lymphocyte ratio.

Given that the follow-up duration in the NHANES 2011 to 2018 dataset was <10 years, we performed time-dependent ROC curve analyses at 1, 3, and 5 years and calculated the corresponding AUCs. During the external validation analyses, the AUCs for MLR in predicting all-cause mortality at 1, 3, and 5 years were 0.68 (95% CI: 0.62–0.73), 0.70 (95% CI: 0.67–0.73), and 0.70 (95% CI: 0.67–0.72), respectively (Fig. S1A and B, Supplemental Digital Content, https://links.lww.com/MD/R667). For cardiovascular mortality, the AUCs at 1, 3, and 5 years were 0.75 (95% CI: 0.65–0.85), 0.75 (95% CI: 0.70–0.80), and 0.73 (95% CI: 0.69–0.77), respectively (Fig. S1C and D, Supplemental Digital Content, https://links.lww.com/MD/R667). In both the primary and external validation analyses, MLR demonstrated moderate predictive ability for both all-cause and cardiovascular mortality among hypertensive patients.

To verify the robustness of our findings, we performed the same sensitivity analyses in the external validation cohort. The results aligned with those from the primary analysis. MLR remained significantly associated with all-cause and cardiovascular mortality across most subgroups, including sex, smoking status, diabetes, MIPA, and anemia (Tables S3 and S4, Supplemental Digital Content, https://links.lww.com/MD/R668). Exceptions were observed in the never-drinking subgroup (*P* = .355 for all-cause mortality; *P* = .151 for cardiovascular mortality) and participants under 60 years of age (*P* = .847 for cardiovascular mortality). A significant interaction between MLR and anemia status was again noted in the all-cause mortality analysis (*P* for interaction = .049). The association between MLR and mortality remained stable after excluding deaths within 3 years of baseline (Table S5, Supplemental Digital Content, https://links.lww.com/MD/R668). Additionally, applying an alternative MLR threshold of 0.8 yielded consistent, statistically significant results (Table S6, Supplemental Digital Content, https://links.lww.com/MD/R668). These findings reinforce the reliability and generalizability of the MLR-mortality association in the hypertensive population.

### 3.7. Comparative analysis of MLR and NLR in predicting all-cause and cardiovascular mortality among individuals with hypertension

To further evaluate the predictive performance of the NLR and the MLR for all-cause and cardiovascular mortality among individuals with hypertension, we constructed Cox proportional hazards models using NLR as the primary independent variable (Table 6), with the same covariate adjustments as those in the MLR-based models. The results showed that the HRs for NLR in relation to all-cause mortality were 1.26 (95% CI: 1.20–1.32), 1.17 (95% CI: 1.13–1.20), and 1.13 (95% CI: 1.08–1.17); and for cardiovascular mortality, the HRs were 1.29 (95% CI: 1.22–1.37), 1.25 (95% CI: 1.18–1.33), and 1.21 (95% CI: 1.13–1.29). The time-dependent AUC values for NLR in predicting 1-, 3-, 5-, and 10-year all-cause mortality were 0.67 (95% CI: 0.61–0.72), 0.65 (95% CI: 0.62–0.68), 0.65 (95% CI: 0.63–0.67), and 0.63 (95% CI: 0.61–0.64), respectively (Fig. S2A and B, Supplemental Digital Content, https://links.lww.com/MD/R667). For cardiovascular mortality, the corresponding AUCs were 0.64 (95% CI: 0.54–0.73), 0.64 (95% CI: 0.60–0.69), 0.67 (95% CI: 0.64–0.70), and 0.64 (95% CI: 0.62–0.66; Fig. S2C and D, Supplemental Digital Content, https://links.lww.com/MD/R667). These findings demonstrate that NLR consistently exhibited lower HRs and AUCs compared to MLR, indicating that MLR may be a superior biomarker for predicting all-cause and cardiovascular mortality in hypertensive individuals. Similarly, external validation using data from the 2011 to 2018 NHANES cohort yielded consistent results (Table S7, Supplemental Digital Content, https://links.lww.com/MD/R668 and Fig. S3, Supplemental Digital Content, https://links.lww.com/MD/R667), further supporting the robustness of our findings.

**Table 6:** Relationship between NLR and mortality in hypertensive patients.

Characteristic	Crude model		Model 1		Model 2	
	HR (95% CI)	*P*	HR (95% CI)	*P*	HR (95% CI)	*P*
All-cause mortality						
NLR	1.26 (1.20–1.32)	<.0001	1.17 (1.13–1.20)	<.0001	1.13 (1.08–1.17)	<.0001
Cardiovascular mortality						
NLR	1.29 (1.22–1.37)	<.0001	1.25 (1.18–1.33)	<.0001	1.21 (1.13–1.29)	<.0001

Crude model was unadjusted for covariates.

Model 1 was adjusted for age, sex, and race.

Model 2 was further adjusted for age, sex, race, marital status, education levels, poverty-income ratio (PIR), body mass index (BMI), total cholesterol (TC), smoking status, drinking status, diabetes, serum creatinine (Scr), blood urea nitrogen (BUN), aspartate aminotransferase (AST), and neutrophils.

CI = confidence interval, HR = hazard ratio, NLR = neutrophil-to-lymphocyte ratio.

## 4. Discussion

This study analyzed NHANES data from 2003 to 2010 to examine the link between MLR and survival outcomes in hypertensive patients. The findings indicate that higher MLR levels correlate with greater all-cause and cardiovascular mortality among these patients. Notably, this association persists even after accounting for various potential confounders.

Hypertension is a multifactorial disease, influenced by various risk factors in its onset and progression. Recent research has shown that the activation of inflammatory and immune responses functions as a critical element in the pathological changes associated with hypertension, contributing not only to its onset and progression but also to its severe complications.^[18]^ Meanwhile, an increasing body of research shows that hypertension is associated with chronic inflammation, marked by the migration, accumulation, and activation of inflammatory cells, along with the release of pro-inflammatory cytokines and free radicals from activated innate immune and endothelial cells.^[19]^ Interleukin (IL)-1 beta, a key member of the IL-1 interleukin family, can upregulate several pro-inflammatory genes, such as IL-6, IL-17, and interferon-gamma; this upregulation can contribute to tissue damage and inflammation-related conditions like hypertension and myocardial infarction.^[20–23]^ Clinical studies have demonstrated a strong link between high plasma IL-6 levels and the severity of resistant hypertension, as well as a 6-year mortality risk.^[24]^ Additionally, other studies have indicated that the activated NLRP3 inflammasome is strongly associated with many chronic inflammatory conditions, such as atherosclerosis and hypertension.^[25]^ These results demonstrate that the occurrence and development of hypertension are significantly linked to inflammatory mediators, providing a theoretical basis for understanding the relationship between the inflammatory marker MLR and the prognosis of hypertension.

Lymphocytes and monocytes are essential for the innate immune system’s defense against pathogen invasion. Lymphocytes are crucial for orchestrating and enhancing adaptive immune responses, with the ability to recognize a wide range of self and non-self-antigens.^[26]^ Inflammatory responses may affect the immune system through various mechanisms, including suppressing lymphocyte activity and leading to decreased lymphocyte levels in the blood.^[27]^ This phenomenon may be attributed to elevated serum cortisol and catecholamine levels during the inflammatory process, which promote lymphocyte apoptosis and inhibit their proliferation and differentiation.^[27,28]^ Interferon-gamma is the sole component of the type II interferon family, and its main producer is type 1 T helper cells. It induces hypertension by increasing angiotensinogen expression in rat kidney tubule cells that are proximal through the Janus kinase 2 /signal transducers and activators of transcription 3-dependent mechanism, thereby increasing blood volume.^[29]^ Consequently, interferon-secreting T cells could create a connection with the renin-angiotensin-aldosterone system by regulating angiotensinogen production, ultimately leading to elevated blood pressure.^[23]^

Monocytes, as crucial components of the innate immune system, not only promote the release of inflammatory mediators and transform into macrophages to participate in immune responses after pathogen invasion, but also play a vital role in cardiovascular health, while serving as primary effector cells in inflammatory processes, playing a key role in microbial clearance and the pathogenesis of inflammatory and degenerative diseases.^[27,30–32]^ Previous studies have confirmed that the number of monocytes is correlated with the severity of atherosclerosis and the extent of myocardial infarction.^[13,33–35]^ In the process of hypertension, a mild inflammatory state that is brought on by endothelial cells, monocytes, and macrophages producing large amounts of hydrogen peroxide and superoxide anion, leads to oxidative stress, vascular dysfunction, and target organ damage.^[23,36]^

There is growing evidence that the MLR can be used as an effective and inexpensive diagnostic tool for various inflammatory and immune diseases, such as bacterial infection-related fever,^[37]^ tuberculosis infection,^[38]^ and infection associatedwith tibial fracture.^[39]^ Compared with other single inflammatory markers such as white blood cell count, monocytes, and lymphocytes, Hua et al believe that combining monocytes and lymphocytes into a single measure more accurately represents systemic inflammation, particularly when their counts approach the extremes of normal ranges.^[27]^ Wang et al consider that the MLR may be more stable than the individual levels of monocytes, lymphocytes, and leukocytes because it reflects the balance between monocyte and lymphocyte levels, making it less affected by various physiological and pathological conditions.^[40]^ It is particularly important that studies have shown the MLR to be a useful indicator for assessing the severity and prognosis of cardiovascular diseases. In the general population, the MLR is a powerful independent predictor of mortality and cardiovascular disease mortality.^[27]^ In patients experiencing non-ST-segment elevation myocardial infarction, the MLR is a standalone risk factor for major adverse cardiac events.^[41]^ Additionally, MLR is independently linked to increased mortality risk in heart failure patients.^[42]^ These findings align with our research, which demonstrates the connection between MLR and both all-cause mortality and cardiovascular death risk in hypertensive individuals. To verify our findings, we performed sensitivity analyses through 3 different methods and an external validation. All results consistently confirmed a significant link between MLR and mortality risk in individuals with hypertension. This robustness underscores the reliability of the MLR as a predictor of mortality risk in hypertensive individuals.

Given the simplicity and cost-effectiveness of MLR measurement, it is of value as a prognostic assessment tool for hypertensive patients in clinical practice. Currently, widely used cardiovascular risk assessment tools, such as the Framingham Risk Score^[43]^ and the Atherosclerotic Cardiovascular Disease risk score,^[44]^ are primarily constructed based on traditional risk factors, including age, sex, blood pressure, lipid levels, and smoking status. However, these scoring systems do not incorporate inflammatory and immune-related markers that play an important role in the development of cardiovascular events. Previous studies have demonstrated that integrating the MLR into the Atherosclerotic Cardiovascular Disease and Framingham risk models can significantly improve the accuracy of cardiovascular mortality prediction.^[45]^ In addition, MLR can be directly calculated from routine complete blood count tests and has notable advantages, including simplicity, wide availability, and low cost, which make it highly feasible for clinical application, particularly in resource-limited healthcare settings. Therefore, further prospective studies are warranted to evaluate the role of MLR in optimizing existing cardiovascular risk stratification models, to provide more accurate and practical tools for clinical risk assessment and individualized decision-making.

Interestingly, our study found that drinking and diabetes are risk factors for all-cause mortality in hypertensive patients, whereas anemia appears to be a protective factor. Excessive alcohol consumption interferes with multiple aspects of immune response, leading to significant deficiencies in the body’s defense against microorganisms, which increases the risk of infectious diseases such as tuberculosis, hepatitis, and pneumonia.^[46]^ Moreover, alcohol consumption is a major risk factor for oral cancer, pharyngeal cancer, esophageal cancer, cardiovascular disease, and liver cirrhosis.^[47,48]^ Importantly, alcohol is strongly correlated with accidents, fights, and violent events,^[49]^ and these factors collectively contribute to a significant increase in the risk of all-cause mortality. Diabetes is closely associated with many serious macrovascular complications (e.g., coronary heart disease and stroke) and microvascular complications (e.g., kidney disease, blindness, and amputation).^[50]^ Moreover, diabetes is associated with higher cancer mortality rates and increased risks of mortality from infectious diseases and respiratory system diseases.^[51]^ Particularly in patients with hypertension combined with diabetes, the incidence and mortality of cardiovascular diseases are higher,^[52]^ which to some extent increases the risk of all-cause mortality. Studies have shown that hemoglobin levels are positively correlated with hypertension, while anemia is negatively correlated with hypertension in adults.^[53]^ Particularly in patients with severe anemia, pathological changes such as decreased blood viscosity and peripheral vasodilation may reduce the risk of cardiovascular events caused by hypertension,^[54]^ thereby lowering all-cause mortality. While this available evidence may partially explain the observed link between anemia and reduced all-cause mortality risk in our study, it is insufficient to validate a definitive biological protective effect of anemia. Our study lacks detailed information on anemia subtypes and related treatments, both of which may have important implications for prognosis.^[55]^ Therefore, this finding warrants cautious interpretation, given that the observed association could be influenced by unmeasured confounding or population-specific traits. Additional clinical cohort and mechanistic studies are required to validate it.

Meanwhile, we also found that MLR outperformed NLR in predicting both all-cause and cardiovascular mortality among individuals with hypertension. Neutrophils are the primary effector cells of acute inflammation and play a critical role in eliminating pathogens during acute immune responses; they are thus considered markers of acute inflammation.^[56,57]^ By contrast, monocytes can infiltrate the subendothelial layer of blood vessel walls, where they differentiate into macrophages, engulf oxidized lipids to form atherosclerotic plaques, and release pro-inflammatory cytokines that drive chronic vascular inflammation.^[58]^ These mechanistic differences may explain why MLR, which reflects the balance between monocyte-driven chronic inflammation and lymphocyte-mediated immune regulation, serves as a more stable and comprehensive biomarker than NLR for long-term mortality risk in hypertensive populations.

However, our study has inevitable limitations. First, as an observational study using NHANES data, we cannot confirm a causal link between elevated MLR and mortality outcomes. Second, despite adjusting for numerous confounding factors, residual confounding cannot be entirely excluded. Specifically, our study lacked information on antihypertensive medication subtypes and specific treatment regimens. Different classes of antihypertensive drugs may have varying effects on inflammatory status and prognosis.^[59]^ Third, our study lacks data on changes in MLR over time, which could provide more information on its prognostic value. Future prospective studies addressing these limitations could further validate the role of MLR in hypertension management.

## 5. Conclusion

This study indicates a significant and independent association between elevated MLR and the risk of all-cause and cardiovascular mortality in hypertensive patients. Notably, MLR exhibits a robust predictive ability for short and long-term mortality. These results suggest that NLR could serve as a simple and convenient tool for identifying high-risk patients and guiding targeted interventions. Further prospective studies are needed to validate these findings and understand the underlying mechanisms.

## Acknowledgments

We thank the patients and investigators who have contributed to the NHANES dataset.

## Author contributions

**Conceptualization:** Qian Zhu, Xuesong Li.

**Funding acquisition:** Qian Zhu, Xuesong Li.

**Formal analysis:** Chao Wang, Bo Li.

**Visualization:** Chao Wang, Bo Li.

**Project administration:** Qikeng Zhang, Zhenyan Xie, Huixi Xie.

**Supervision:** Qian Zhu, Xuesong Li.

**Writing – original draft:** Chao Wang, Yun Gao.

**Writing – review & editing:** Qian Zhu, Xuesong Li.

## Supplementary Material




