# Supplementary material for: Elevated monocyte-lymphocyte ratio as an independent predictor of all-cause and cardiovascular mortality in hypertensive patients: A retrospective cohort study
Source: Medicine (Baltimore). 2026 Apr 10;105(15):e48243. doi: 10.1097/MD.0000000000048243 (PMC13593092; doi:10.1097/MD.0000000000048243)
Supplement: Supplementary file 1 [file medi-105-e48243-s001.pdf]

Supplementary Table S1. Sensitivity analysis of the association between MLR and all-cause and cardiovascular mortality in hypertensive patients: excluding those who died within 3 years

Characteristic	Crude Model		Model1		Model2	
	HR (95% CI)	*P*	HR (95% CI)	*P*	HR (95% CI)	*P*
All-cause mortality						
MLR	17.22(11.57,25.62)	<0.0001	3.36(2.55,4.41)	<0.0001	3.06(2.25,4.17)	<0.0001
MLR category						
Lower MLR	ref	ref	ref	ref	ref	ref
Higher MLR	2.94(2.67,3.24)	<0.0001	1.45(1.31,1.61)	<0.0001	1.37(1.23,1.52)	<0.0001
Cerebrovascular mortality						
MLR	26.15(15.45,44.27)	<0.0001	5.85(4.02,8.53)	<0.0001	5.64(3.54,8.99)	<0.0001
MLR category						
Lower MLR	ref	ref	ref	ref	ref	ref
Higher MLR	4.02(3.40,4.76)	<0.0001	1.71(1.45,2.02)	<0.0001	1.58(1.33,1.88)	<0.0001

Crude model was unadjusted for covariates.

Model 1 was adjusted for age, sex, and race.

Model 2 was further adjusted for age, sex, race, marital status, education levels, PIR, BMI, TC, smoking status, drinking status, diabetes, Scr, BUN, AST, and neutrophils.

Supplementary Table S2. Sensitivity analysis of the association between MLR (optimal cutoff value = 0.8) and all-cause and cardiovascular mortality in hypertensive patients

Characteristic	Crude Model		Model1		Model2	
	HR (95% CI)	*P*	HR (95% CI)	*P*	HR (95% CI)	*P*
All-cause mortality						
MLR	16.69(11.24,24.77)	<0.0001	3.57(2.83,4.51)	<0.0001	3.24(2.53,4.14)	<0.0001
MLR category						
Lower MLR	ref	ref	ref	ref	ref	ref
Higher MLR	6.88(4.71,10.04)	<0.0001	3.35(2.44,4.60)	<0.0001	3.13(2.08,4.71)	<0.0001
Cerebrovascular mortality						
MLR	23.91(14.39,39.73)	<0.0001	5.54(3.97,7.72)	<0.0001	5.49(3.84,7.86)	<0.0001
MLR category						
Lower MLR	ref	ref	ref	ref	ref	ref
Higher MLR	13.05(7.88,21.61)	<0.0001	5.51(4.11,7.39)	<0.0001	4.53(2.89,7.10)	<0.0001

Crude model was unadjusted for covariates.

Model 1 was adjusted for age, sex, and race.

Model 2 was further adjusted for age, sex, race, marital status, education levels, PIR, BMI, TC, smoking status, drinking status, diabetes, Scr, BUN, AST, and neutrophils.

Supplementary Table S3. External validation of associations between MLR and all-cause mortality across subgroups in hypertensive patients

Characteristics	Lower MLR (≤0.39)	Higher MLR HR (95% CI)	*P*	*P* for interaction
Age, y				0.354
<60	1	2.372(1.457,3.863)	<0.001	
≥60	1	1.690(1.316,2.169)	<0.0001	
Sex				0.978
Female	1	2.308(1.667,3.196)	<0.0001	
Male	1	2.306(1.708,3.113)	<0.0001	
Smoking status				0.296
Never	1	2.298(1.824,2.896)	<0.0001	
Former/Now	1	2.472(1.817,3.364)	<0.0001	
Drinking status				0.127
Never	1	1.325(0.730,2.405)	0.355	
Former/mild/ moderate/heavy	1	2.559(2.085,3.141)	<0.0001	
Diabetes				0.154
No	1	2.507(1.996,3.148)	<0.0001	
Yes	1	2.061(1.400,3.035)	<0.001	
MIPA				0.542

Supplementary Table S3. External validation of associations between MLR and all-cause mortality across subgroups in hypertensive patients (continued)

Characteristics	Lower MLR (≤0.39)	Higher MLR HR (95% CI)	*P*	*P* for interaction
No	1	2.232(1.725,2.887)	<0.0001	
Yes	1	2.705(1.800,4.067)	<0.0001	
Anemia				0.049
No	1	2.465(1.944,3.127)	<0.0001	
Yes	1	1.712(1.133,2.586)	0.011	

HRs were adjusted for adjusted for age, sex, race, marital status, education levels, PIR, BMI, TC, smoking status, drinking status, diabetes, Scr, BUN, AST, and neutrophils.

Supplementary Table S4. External validation of associations between MLR and cardiovascular mortality across subgroups in hypertensive patients

Characteristics	Lower MLR (≤0.39)	Higher MLR HR (95% CI)	*P*	*P* for interaction
Age, y				0.172
<60	1	1.110(0.385, 3.205)	0.847	
≥60	1	2.205(1.504,3.233)	<0.0001	
Sex				0.474
Female	1	2.484(1.549,3.982)	<0.001	
Male	1	3.095(1.812,5.289)	<0.0001	
Smoking status				0.298
Never	1	2.867(1.962,4.190)	<0.0001	
Former/Now	1	3.257(1.969, 5.389)	<0.0001	
Drinking status				0.422
Never	1	1.961(0.782, 4.916)	0.151	
Former/mild/ moderate/heavy	1	3.253(2.322,4.558)	<0.0001	
Diabetes				0.341
No	1	2.974(1.802, 4.909)	<0.0001	
Yes	1	2.903(1.694,4.975)	<0.001	
MIPA				0.585

Supplementary Table S4. External validation of associations between MLR and cardiovascular mortality across subgroups in hypertensive patients (continued)

Characteristics	Lower MLR (≤0.39)	Higher MLR HR (95% CI)	*P*	*P* for interaction
No	1	3.131(2.126,4.610)	<0.0001	
Yes	1	2.423(1.283,4.575)	0.006	
Anemia				0.678
No	1	2.874(1.944,4.249)	<0.0001	
Yes	1	2.600(1.212,5.579)	0.014	

HRs were adjusted for adjusted for age, sex, race, marital status, education levels, PIR, BMI, TC, smoking status, drinking status, diabetes, Scr, BUN, AST, and neutrophils.

Supplementary Table S5. External validation analysis of the association between MLR and all-cause and cardiovascular mortality in hypertensive participants: excluding who died within 3 years

Characteristic	Crude Model		Model1		Model2	
	HR (95% CI)	*P*	HR (95% CI)	*P*	HR (95% CI)	*P*
All-cause mortality						
MLR	7.54(3.65,15.58)	<0.0001	3.21(1.93,5.31)	<0.0001	2.97(1.84,4.77)	<0.0001
MLR category						
Lower MLR	ref	ref	ref	ref	ref	ref
Higher MLR	3.19(2.57,3.95)	<0.0001	1.49(1.18,1.88)	<0.001	1.35(1.07,1.71)	0.01
Cerebrovascular mortality						
MLR	10.08(4.67,21.76)	<0.0001	5.53(3.01,10.16)	<0.0001	6.76(3.60,12.68)	<0.0001
MLR category						
Lower MLR	ref	ref	ref	ref	ref	ref
Higher MLR	4.61(3.25,6.53)	<0.0001	1.91(1.32,2.77)	<0.001	1.72(1.17,2.51)	0.01

Crude model was unadjusted for covariates.

Model 1 was adjusted for age, sex, and race.

Model 2 was further adjusted for age, sex, race, marital status, education levels, PIR, BMI, TC, smoking status, drinking status, diabetes, Scr, BUN, AST, and neutrophils.

Supplementary Table S6. External validation analysis of the relationship between MLR (optimal cutoff value = 0.8) and all-cause and cardiovascular mortality among hypertensive patients

Characteristic	Crude Model		Model1		Model2	
	HR (95% CI)	*P*	HR (95% CI)	*P*	HR (95% CI)	*P*
All-cause mortality						
MLR	9.02(4.63,17.56)	<0.0001	3.84(2.54,5.80)	<0.0001	3.54(2.51,4.98)	<0.0001
MLR category						
Lower MLR	ref	ref	ref	ref	ref	ref
Higher MLR	8.36(5.81,12.02)	<0.0001	3.02(2.14,4.26)	<0.0001	2.24(1.49,3.37)	<0.001
Cardiovascular mortality						
MLR	11.54(5.32,25.03)	<0.0001	5.75(3.26,10.13)	<0.0001	6.43(3.82,10.85)	<0.0001
MLR category						
Lower MLR	ref	ref	ref	ref	ref	ref
Higher MLR	14.77(8.37,26.07)	<0.0001	4.76(2.63,8.62)	<0.0001	4.39(2.38,8.10)	<0.0001

Crude model was unadjusted for covariates.

Model 1 was adjusted for age, sex, and race.

Model 2 was further adjusted for age, sex, race, marital status, education levels, PIR, BMI, TC, smoking status, drinking status, diabetes, Scr, BUN, AST, and neutrophils.

Supplementary Table S7. External validation analysis of the association between NLR and mortality in hypertensive patients.

Characteristic	Crude Model		Model 1		Model 2	
	HR (95% CI)	*P*	HR (95% CI)	*P*	HR (95% CI)	*P*
All-cause mortality						
NLR	1.20(1.14,1.26)	<0.0001	1.10(1.06,1.15)	<0.0001	1.06(1.02,1.11)	0.004
Cardiovascular mortality						
NLR	1.22(1.15,1.30)	<0.0001	1.11(1.05,1.17)	<0.001	1.06(1.00,1.13)	0.05

Crude model was unadjusted for covariates.

Model 1 was adjusted for age, sex, and race.

Model 2 was further adjusted for age, sex, race, marital status, education levels, PIR, BMI, TC, smoking status, drinking status, diabetes, Scr, BUN, AST, and neutrophils.
