# Supplementary material for: Elevated monocyte-lymphocyte ratio as an independent predictor of all-cause and cardiovascular mortality in hypertensive patients: A retrospective cohort study
Source: Medicine (Baltimore). 2026 Apr 10;105(15):e48243. doi: 10.1097/MD.0000000000048243 (PMC13593092; doi:10.1097/MD.0000000000048243)
Supplement: Supplementary file 2 [file medi-105-e48243-s002.pdf]

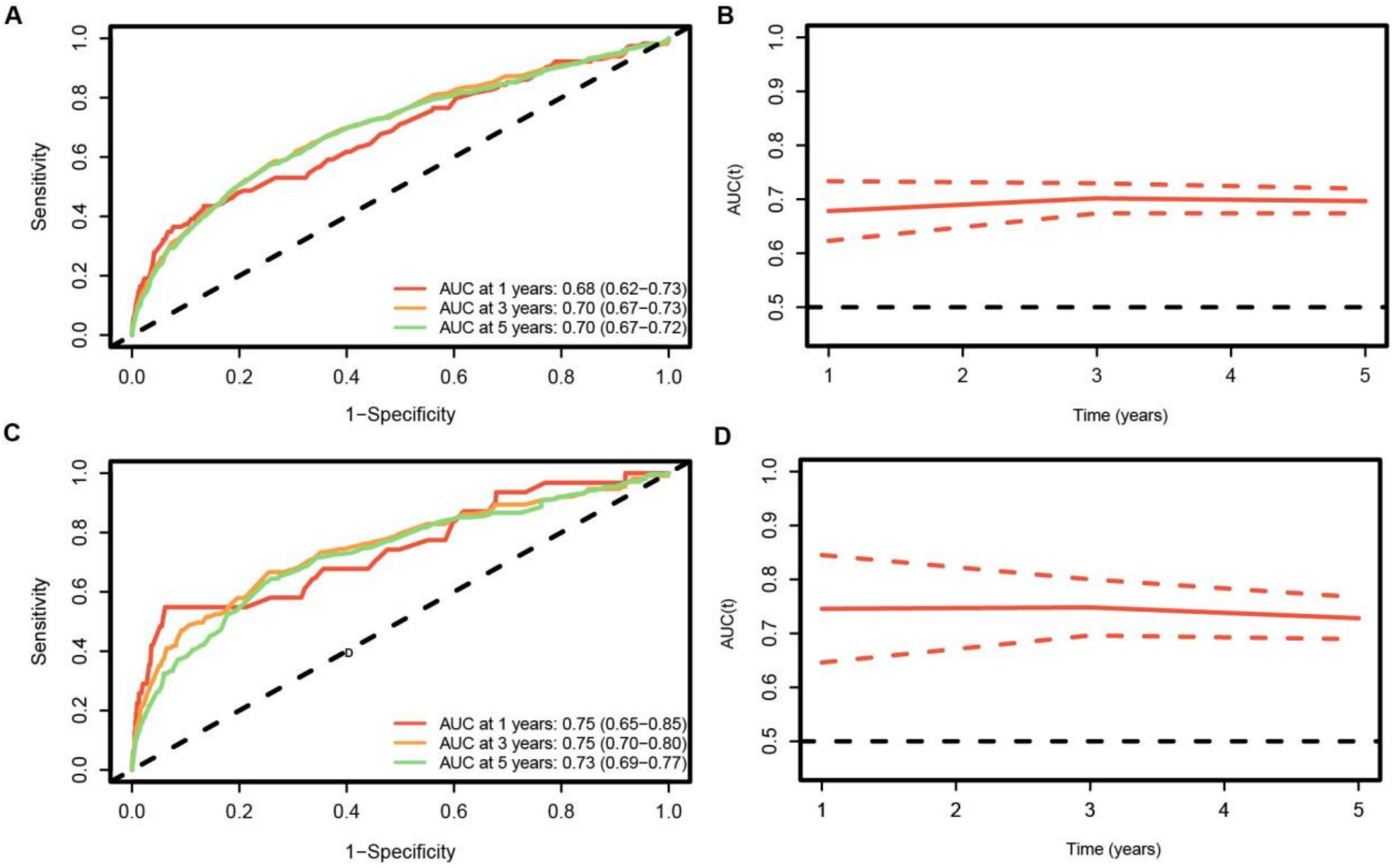

Supplementary Figure S1. External validation analysis of time-dependent ROC curves and time-dependent AUC values of the MLR for predicting all-cause mortality (**A, B**) and cardiovascular mortality (**C, D**).

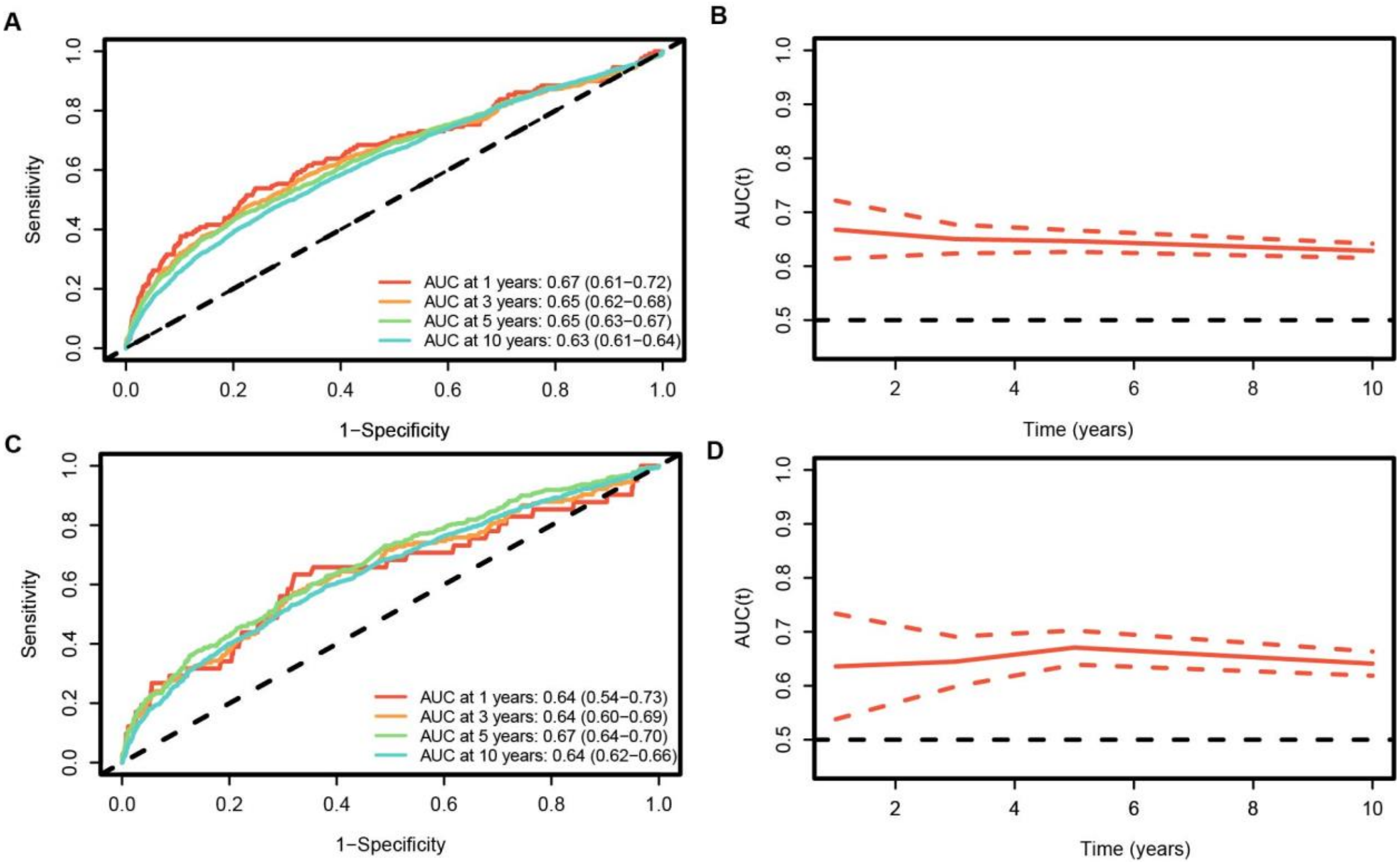

Supplementary Figure S2. Time-dependent ROC curves and time-dependent AUC values of the NLR for predicting all-cause mortality (**A, B**) and cardiovascular mortality (**C, D**).

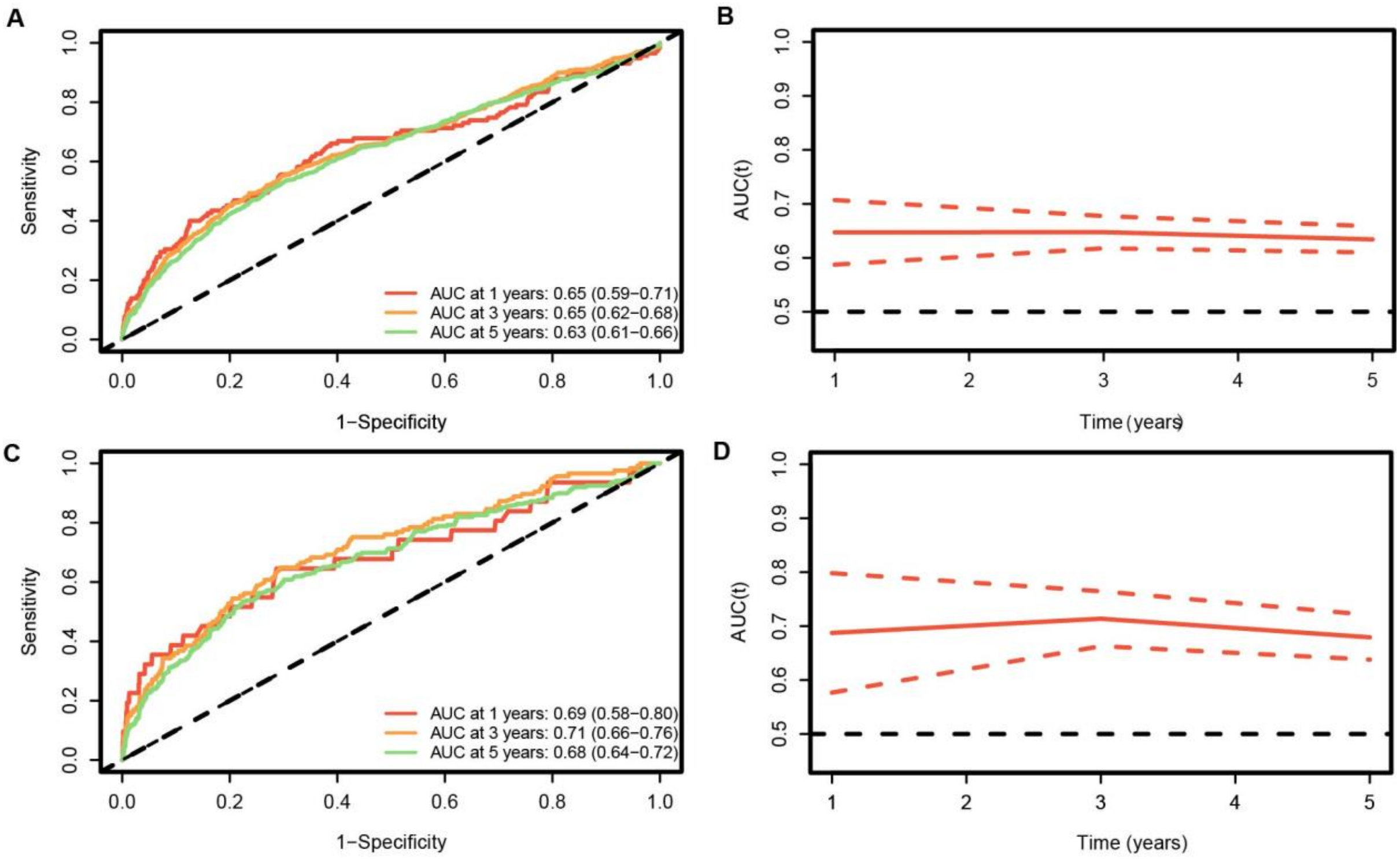

Supplementary Figure S3. External validation analysis of time-dependent ROC curves and time-dependent AUC values of the NLR for predicting all-cause mortality (**A, B**) and cardiovascular mortality (**C, D**).
